# Polygenic Risk for Schizophrenia, Brain Structure, and Environmental Risk in UK Biobank

**DOI:** 10.1093/schizbullopen/sgab042

**Published:** 2021-09-24

**Authors:** Xingxing Zhu, Joey Ward, Breda Cullen, Donald M Lyall, Rona J Strawbridge, Daniel J Smith, Laura M Lyall

**Affiliations:** 1 Institute of Health and Wellbeing, University of Glasgow, Glasgow, UK; 2 Cardiovascular Medicine Unit, Department of Medicine Solna, Karolinska Institutet, Stockholm, Sweden; 3 Health Data Research (HDR), Glasgow, UK; 4 Centre for Clinical Brain Sciences, University of Edinburgh, Edinburgh, UK

**Keywords:** schizophrenia, polygenic risk, environmental risk, cortical thickness, surface area, cortical volume

## Abstract

Schizophrenia is a heritable neurodevelopmental disorder characterized by neuroanatomical changes in the brain, but exactly how increased genetic burden for schizophrenia influences brain structure is unknown. Similarly, how environmental risk factors for schizophrenia impact brain structure is not fully understood. Here we investigated how genetic burden for schizophrenia (indexed by a polygenic risk score, PRS-SCZ) was associated with cortical thickness (CT), surface area (SA), cortical volume (CV), and subcortical structures within 18 088 White British ancestry participants with derived brain phenotypes from UK Biobank. We also explored whether environmental risk factors for schizophrenia (childhood trauma, cannabis use, birth weight, season of birth, and Townsend social deprivation index) exacerbated the impact of PRS-SCZ on brain structure. We found that PRS-SCZ was associated with lower CT in the frontal lobe, insula lobe, lateral orbitofrontal cortex, medial orbitofrontal cortex, posterior cingulate cortex, and inferior frontal cortex, and reduced SA and CV in the supramarginal and superior temporal cortex, but not with subcortical volumes. When models included environmental risk factors as covariates, PRS-SCZ was only associated with lower SA/CV within the supramarginal cortex, superior temporal cortex, and inferior frontal cortex. Moreover, no interactions were observed between PRS-SCZ and each of the environmental risk factors on brain structure. Overall, we identified brain structural correlates of PRS-SCZ predominantly within frontal and temporal regions and some of these associations were independent of environmental risk factors, suggesting that they may represent vulnerable biomarkers of genetic risk for schizophrenia. Future research is warranted to establish these associations beyond older White British individuals.

## Introduction

Schizophrenia is a debilitating and complex psychiatric disorder, affecting about 1% of the population.^1^ It is a heritable neurodevelopmental condition that arises via a complex interaction of genetic and environmental risk factors.^2^ Neuroimaging studies have reported brain structural alterations in cortical and subcortical regions in people with schizophrenia relative to healthy individuals.^3–6^ These brain changes could be driven by genetic factors and may mediate the effect of genetic risk on the phenotype of schizophrenia.^7–9^ Genome-wide association studies (GWAS) have identified hundreds of significant loci and indicate a polygenic architecture of schizophrenia.^8,10,11^ A polygenic risk score for schizophrenia, which captures the cumulative effect of significant risk loci across the whole genome, is useful in exploring the neurobiology of schizophrenia.^12–14^ To date, little is known about how polygenic risk for schizophrenia influences brain structure, or how environmental risk factors may influence these associations.

Convergent evidence has demonstrated widespread gray matter reductions in individuals with schizophrenia, including lower cortical volume (CV), reduced cortical thickness (CT), and smaller surface area (SA) and subcortical volumes, predominantly in the frontal and temporal regions.^3,15–17^ Longitudinal neuroimaging studies have demonstrated progressive gray matter changes related to schizophrenia^18–20^ as well as morphological differences that may be present prior to the onset of symptoms.^20,21^ High-risk familial studies have also observed brain structural differences in unaffected relatives of patients with schizophrenia relative to controls, suggesting a genetic contribution.^22–24^ The association between polygenic risk scores for schizophrenia (PRS-SCZ) and brain structure has been the subject of some early investigations. Initial work focused on total brain volume, total gray/white matter volume, lateral ventricular volume, and subcortical volumes,^25,26^ and a systematic review of seven studies found inconsistent findings plus a lack of significant associations.^27^ Recent studies with larger samples reported negative associations with volume in the pallidum, thalamus, and hippocampus, as well as positive associations with the caudate and putamen.^13,28^ Studies on cortical measures have reported negative associations between PRS-SCZ and global CT and insular lobe CT^12^, frontal and temporal cortex CT^5^, rostral anterior cingulate cortex CT^29^ within the general population, as well as global cortical thinning in patients with schizophrenia and bipolar disorder.^30^ Stauffer et al.^28^ investigated both micro- and macro-structural MRI metrics, and found colocalization of polygenic risk effects was in frontal cortex, insular, medial and lateral temporal cortex, and ventral visual stream. Relatively few studies have systematically examined associations between PRS-SCZ and regional CT, SA, and CV.^5,12,29^ In addition, almost all published studies in this area have not taken account of the possible influence of environmental risk factors for schizophrenia on brain structure.^5,12,28,29,31^

Environmental risk factors, along with their interaction with genetic risk, contribute to the occurrence and development of schizophrenia.^2,32,33^ Epidemiological studies have identified multiple risk factors for psychotic disorders, of which childhood trauma, cannabis use, low socioeconomic status, obstetric complications, winter/spring season of birth, and non-right handedness play important roles.^34–39^ In addition, previous studies have found significant interactions between PRS-SCZ and cannabis use, childhood trauma^40^ and birth weight,^41^ but not for winter birth,^40^ despite inconsistent results being reported.^42,43^ Furthermore, environmental risk factors may contribute, at least in part, to some of the brain structural abnormalities observed in schizophrenia^30,44,45^ and may also contribute to gene-environment interactions.^45,46^ Habets et al.^46^ reported stronger reductions of CT for exposure to trauma and cannabis in patients with schizophrenia (vs. healthy controls/ siblings); Cancel et al.^47^ found greater reduction of total grey matter in patients who had been exposed to emotional neglect; French et al.^45^ observed more longitudinal reduction in mean CT in adolescents who had ever used cannabis (vs. never used). These studies support the idea that there may be interactions between genetic risk for schizophrenia and environmental risk in contributing to brain abnormalities related to schizophrenia.

Here we investigate associations between PRS-SCZ and cortical metrics (CV, SA, and CT), as well as subcortical volumes, using data from ~18 000 White British ancestry individuals from UK Biobank. We also assess the impact of environmental risk factors (childhood trauma, cannabis use, birth weight, winter/spring season of birth, and Townsend social deprivation index) on any observed associations between PRS-SCZ and these environmental risk factors. We hypothesized that a higher PRS-SCZ would be associated with lower global CT, thinner frontal and temporal cortices, thinner insula lobe, smaller thalamus, pallidum, and hippocampus.^5,28,48,49^ In addition, we expected that environmental risk factors would influence the associations between PRS-SCZ and related brain structural differences.

## Methods

### Participants

This study was conducted using participants from UK Biobank under approval from the NHS National Research Ethics Service (UK Biobank-approved applications #6553 and #17689). Freesurfer data (released by Jan 2020) based on the Desikan-Killiany cortical atlas^50^ were available for a total of 21 915 participants. Participants were then excluded for the following reasons: having a developmental or neurological disorder (See Supplementary Table S1 for detailed participant exclusion criteria); self-reported schizophrenia; having withdrawn from the UK Biobank project; non-White British ancestry based on self-report and genetic data; genetic data failing to pass quality control and having an intracranial volume or a PRS-SCZ beyond three standard deviations from the sample mean.

### Derivation of the Polygenic Risk Score for Schizophrenia

LDpred was utilized to calculate the PRS-SCZ,^51^ based on the summary statistics from a recent GWAS specific to schizophrenia,^10^ which included 33 426 schizophrenia patients and 54 065 controls, excluding individuals from UK Biobank. Participants were excluded if over 10% of genetic data was missing; if self-reported sex did not match genetic sex; if purported sex chromosome aneuploidy was reported; if heterozygosity value was a clear outlier and if the PRS-SCZ was beyond three standard deviations from the sample mean. Finally, a total of 18 088 participants were included in this study.

### Brain Imaging Variables

All neuroimaging data were acquired, pre-processed, quality controlled, and made available by UK Biobank (https://biobank.ctsu.ox.ac.uk/crystal/crystal/docs/brain_mri.pdf). Details on the acquisition parameters and the imaging protocol are documented online and are described within the protocol paper.^52^ Derived phenotypes were used in this study. The main neuroimaging data consisted of global, lobar, and regional values for CT, SA, and CV of 33 regions (data of temporal pole are not available) in each hemisphere from the Desikan-Killiany cortical atlas.^50^ Global and lobar values were calculated as per Neilson et al.^12^ In addition, we analyzed subcortical volumes including thalamus, caudate, putamen, pallidum, hippocampus, amygdala, accumbens processed by subcortical volumetric segmentation in Freesurfer. A more detailed description of these variables is provided within Supplementary Material.

### Environmental Risk Factors

Childhood trauma was assessed using the five-item Childhood Trauma Screener developed by Glaesmer et al .^53^ These questions cover the self-reported frequency of feeling loved, being physically abused, feeling hated, being sexually molested, and being taken to the doctor when needed as a child (data field 20487 to 20491). Participants answered these questions by selecting “Prefer not to answer,” “Never true,” “Rarely true,” “Sometimes true,” “Often,” or “Very often true” (Supplementary Material).

Cannabis use (https://biobank.ctsu.ox.ac.uk/crystal/field.cgi?id=20453) was reported via an online question, “Have you taken CANNABIS (marijuana, grass, hash, ganja, blow, draw, skunk, weed, spliff, dope), even if it was a long time ago?.” Participants chose from “Prefer not to answer,” “No,” “Yes, 1–2 times,” “Yes, 3–10 times,” “Yes, 11–100 times,” and “Yes, more than 100 times.”

Birth weight (https://biobank.ctsu.ox.ac.uk/crystal/field.cgi?id=20022) was reported by participants during a verbal interview. To maximize the number of participants included in analyses, missing values at the initial assessment visit were replaced by those at the first repeat assessment visit or the imaging visit.

Winter/spring season of birth in Northern hemisphere (December to May) was obtained from the month of birth in baseline population characteristics (https://biobank.ndph.ox.ac.uk/showcase/field.cgi?id=52).

The Townsend deprivation index (https://biobank.ctsu.ox.ac.uk/crystal/field.cgi?id=189) is based on postcode reported at baseline assessment in UK Biobank. A greater Townsend index score implies a higher level of neighborhood social and economic deprivation.

### Statistical Analysis

All analyses were carried out using Stata/MP 15.0. For associations between PRS-SCZ and brain structure, PRS-SCZ was set as the independent variable; each brain structural measure was set as an outcome. PRS-SCZ × hemisphere interactions were examined in a repeated measures format to determine whether analysis of left and right homologous structures separately was required, with sex, age, age,^2^ hemisphere, ICV, scanner positions on the x, y, and z axes, genotype array and the first fifteen genetic principal components included as covariates. For brain measures that did not show PRS-SCZ × hemisphere interaction, we repeated the linear mixed model with hemisphere as a fixed effect and included the same covariates as in analyses testing for interaction effects. If there was a significant interaction, analyses on both lateralized structures would be conducted separately. PRS-SCZ and each brain outcome were rescaled into zero mean and unitary standard deviation. Participants with each brain outcome beyond three standard deviations were excluded. False Discovery Rate (FDR) correction,^54^ with a significance threshold of *P* < .05, was applied for each metric individually, by correcting over all eight possible lobar structures, twenty-six parcellations, or seven subcortical volumes, using “p.adjust” function in R.

Furthermore, considering the potential impact of environmental risk factors, we investigated whether associations between PRS-SCZ and brain measures were significant when childhood trauma, cannabis use, birth weight, season of birth, and Townsend deprivation index were included as additional covariates. In total, 8667 individuals had complete data for all five environmental factors. Because the subsample was much smaller compared to that in the main analyses, we wished to clarify whether the reduction in sample size influenced the results. Therefore, we repeated previous analyses in this subsample (*N* = 8667) without controlling for environmental risk factors. For further examination of the influence of environmental risk factors on the relationship between PRS-SCZ and brain structure, we excluded 21 individuals who had used antipsychotic medication (self-report at the imaging visit), considering that antipsychotic medication may also influence brain structure.^55,56^

We also examined the interaction between PRS-SCZ and each of the environmental risk factors on brain structure. The interaction term, together with main effects of the PRS-SCZ and the environmental risk factor were included in the model, as well as the same covariates in the analysis for PRS-SCZ and brain structure. Multiple comparisons correction for interaction effects was done using FDR correction (*P* < .05) in the same way.

## Results

### Demographics

A total of 18,088 participants (ages 45–80 years, 8650 males) were included in the analysis. Characteristics of the participants are provided in Table 1. There was no significant sex difference in PRS-SCZ (female = –0.52, male = –0.52, *t* = 0.955, *P* = .340), but the differences in birth weight (female = 3.28 kg, male = 3.46 kg, *t* = –16.519, *P* < .001), childhood trauma (female = 1.79, male = 1.55, *t* = 6.067, *P* < .001) and cannabis use (female = 0.33, male = 0.49, *t* = –10.152, *P* < .001) were significant.

**Table 1.** Descriptive Statistics for Demographic Variables (*N* = 18 088)

Measure	N (%) or Mean ± SD (range)
Gender	Male: 8621 (47.66%)
Age (Years)	62.78 ± 7.46 (45–80)
Polygenic Risk Score	–0.520 ± 0.152 (–0.985 to –0.057)
Self-Reported Mental Health Status	Depression/ postnatal depression: 1513 (8.36%)
	Mania/bipolar disorder/manic depression: 38 (0.21%)
	Other mental disorders: 388 (2.15%)
Winter/Spring Season of Birth	Yes: 9201 (50.87%)
Townsend Deprivation Index at Recruitment (*N* = 18 071)	–2.11 ± 2.57 (–6.26 to 9.16)
Birth Weight (Kg; *N* = 11 921)	3.36 ± 0.61 (0.74–6.78)
Childhood Trauma (*N* = 13 001)	1.68 ± 2.32 (0–20)
Antipsychotic Medication (*N* = 17 176)	No: 17 138 (99.78%)
	Yes: 38 (0.22%)
Cannabis Use (*N* = 13 253)	No: 10 520 (79.38%)
	Yes, 1–2 times: 1227 (9.26%)
	Yes, 3–10 times: 716 (5.40%)
	Yes, 11–100 times: 497 (3.75%)
	Yes, more than 100 times: 293 (2.21%)

*Note:* Other mental disorders: post-traumatic stress disorder, anorexia/bulimia/other eating disorder, obsessive compulsive disorder, anxiety/panic attacks.

### Associations Between PRS-SCZ and Cortical Thickness

We found a significant interaction between PRS-SCZ and hemisphere on the CT in the occipital lobe (Supplementary Table S2), thus analyses on the CT in the left and right occipital lobes were additionally performed. There was no significant association between PRS-SCZ and global CT (β =  –0.012, *P*_*corrected*_ = .237; Table 2 shows significant associations only; Supplementary Table S3 lists all the results). In the eight lobar structures, CT in the frontal lobe (β = –0.018, *P*_*corrected*_ = .050, *R*^*2*^ = 0.006%; Table 2) and insula lobe (β = –0.016, *P*_*corrected*_ = .050, *R*^*2*^ = 0.010%) were associated with PRS-SCZ. Moreover, we found a higher PRS-SCZ was associated with reduced CT in the lateral orbitofrontal cortex (β = –0.025, *P*_*corrected*_ < .001, *R*^*2*^ = 0.018%), the medial orbitofrontal cortex (β = –0.019, *P*_*corrected*_ = .013, *R*^*2*^ = 0.013%), the posterior cingulate cortex (β = –0.021, *P*_*corrected*_ = .009, *R*^*2*^ = 0.020%) and the inferior frontal cortex (β = –0.026, *P*_*corrected*_ < .001, *R*^*2*^ = 0.015%). Figure 1A illustrates the associations between PRS-SCZ and regional CT. In the analyses excluding participants with mental disorders, the results were equivalent (Supplementary Table S4).

**Table 2.** The Association Between Polygenic Risk Score for Schizophrenia and Brain Structures

Brain Structure	N	β	SE	z	P	*P*_*corrected*_	*R* ^*2*^
***Lobes***							
***Cortical Thickness***							
Frontal Lobe	18 035	–0.018	0.007	–2.75	.006	.050	0.006
Insula Lobe	18 085	–0.016	0.006	–2.59	.010	.050	0.010
***Parcellations***							
***Cortical Thickness***							
Lateral Orbitofrontal	18 071	–0.025	0.007	–3.83	<.001	<.001	0.018
Medial Orbitofrontal	18 073	–0.019	0.006	–3.03	.002	.013	0.013
Posterior Cingulate	18 084	–0.021	0.006	–3.46	.001	.009	0.020
Inferior Frontal	18 037	–0.026	0.006	–4.09	<.001	<.001	0.015
***Surface Area***							
Supramarginal	18 072	–0.016	0.005	–3.15	.002	.026	0.017
Superior Temporal	18 073	–0.015	0.005	–3.16	.002	.026	0.012
***Cortical Volume***							
Supramarginal	18 073	–0.016	0.005	–3.19	.001	.013	0.017
Superior Temporal	18 061	–0.018	0.005	–3.59	<.001	<.001	0.015

*Note:* SE, standard error; *R*^*2*^, estimate of variance explained by PRS in %.

**Fig. 1.**
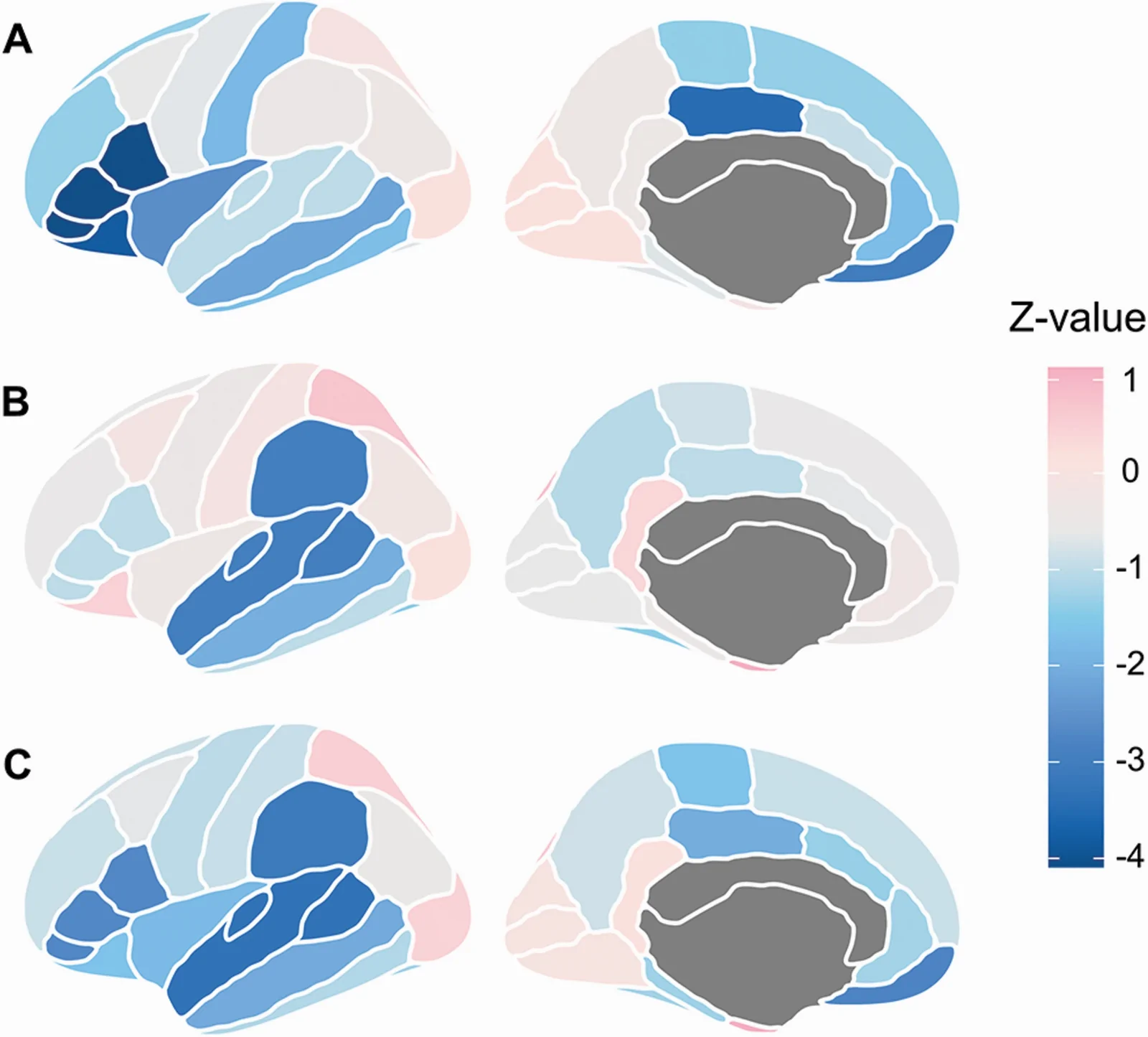
Cortical map of associations between the polygenic risk score for schizophrenia and (A) cortical thickness, (B) surface area and (C) cortical volume. Regions were mapped on the left hemisphere. Pink colours indicate positive associations and blue colours negative associations.

In the subsample with complete data on environmental risk factors (childhood trauma, cannabis use, birth weight, winter/spring season of birth, and Townsend deprivation index), PRS-SCZ was modestly associated with thinner CT in the above regions (Supplementary Table S5), although none of these associations were significant after FDR correction in this smaller sample. Similarly, after controlling for the environmental risk factors and excluding participants who had used antipsychotic medication, there were no significant associations, although a similar negative pattern of association was found (Table 3 shows significant associations only; Supplementary Table S6 lists all the results; Supplementary Figure S1).

**Table 3.** The Association Between Polygenic Risk Score for Schizophrenia and Brain Structures, Adjusted for Childhood Trauma, Cannabis Use, Birth Weight, Winter/Spring Season of Birth, Townsend Deprivation Index and Excluding Participants Having used Antipsychotics

Brain Structure	N	β	SE	z	P	*P*_*corrected*_
***Surface Area***						
Supramarginal	8638	–0.028	0.007	–3.93	<.001	<.001
Superior Temporal	8637	–0.021	0.007	–3.06	.002	.026
Inferior Frontal	8639	–0.022	0.008	–2.86	.004	.035
***Cortical Volume***						
Inferior Frontal	8635	–0.025	0.008	–3.29	.001	.026

### Associations Between PRS-SCZ and Surface Area

No interactions were observed between PRS-SCZ and hemisphere for global or regional SA (Supplementary Table S2). Although PRS-SCZ was not associated with global SA (β = –0.002, *P*_*corrected*_ = .665; Supplementary Table S3; Figure 1B), we found an association with reduced SA in the supramarginal cortex (β = –0.016, *P*_*corrected*_ = .026, *R*^*2*^ = 0.017%; Table 2) and the superior temporal cortex (β = –0.015, *P*_*corrected*_ = .026, *R*^*2*^ = 0.012%). In the analyses including healthy participants only, we found the same results (Supplementary Table S4). Analyses in the subsample also found significant associations with these two regions, in addition to inferior frontal cortex (Supplementary Table S5).

When these analyses were adjusted for environmental factors, the associations with supramarginal cortex (β = –0.028, *P*_*corrected*_ < .001; Table 3), superior temporal cortex (β = –0.021, *P*_*corrected*_ = .026) and inferior frontal cortex (β = –0.022, *P*_*corrected*_ = .035) remained significant.

### Associations Between PRS-SCZ and Cortical Volume

Analyses for the interaction between PRS-SCZ and hemisphere on global or regional CV did not demonstrate any significant effects (Supplementary Table S2). Similar to the results for surface area, PRS-SCZ was not associated with global CV (β = –0.004, *P*_*corrected*_ = .323; Supplementary Table S3; Figure 1C), but supramarginal cortex (β = –0.016, *P*_*corrected*_ = .013, *R*^*2*^ = 0.017%; Table 2) and superior temporal cortex (β = –0.018, *P*_*corrected*_ < .001, *R*^*2*^ = 0.015%). Analyses including only participants without self-reported mental illness found the same results (Supplementary Table S4).

Analyses in the subsample found associations with the supramarginal cortex, superior temporal cortex, and inferior frontal cortex (Supplementary Table S5). Among them, inferior frontal cortex (β = –0.026, *P*_*corrected*_ = .026; Table 3) remained significant when environmental risk factors were added as covariates.

### Associations Between PRS-SCZ and Subcortical Volumes

Analyses for the interaction between PRS-SCZ and hemisphere found a significant interaction effect for the hippocampus (β = 0.011, *P*_*corrected*_ = .025; Supplementary Table S2) and nucleus accumbens (β = –0.015, *P*_*corrected*_ = .025). Therefore, we also examined the left and right hippocampus and nucleus accumbens separately in the following analyses.

PRS-SCZ was associated with reduced volume in the right accumbens before (β = –0.013, *P*_*uncorrected*_ = .043; Supplementary Table S3), but not after FDR correction. Similarly, when environmental risk factors were taken into account, no subcortical structure survived correction for multiple comparisons (Supplementary Table S5).

### PRS-SCZ and Environmental Risk Factors

We firstly tested gene by environment correlation using the linear regression model for each of the environmental factors. We found PRS-SCZ was significantly associated with Townsend deprivation index (β = 0.015, *t* = 1.99, *P* = .047), cannabis use (β = 0.028, *t* =3.28, *P* = .001) and childhood trauma (β = 0.060, *t* = 6.66, *P* < .001), but not birth weight (β = –0.006, *t* = –0.62, *P* = .535).

For the interaction between PRS-SCZ and each risk factor, we found no statistically significant results, albeit some modest associations before FDR correction. For example, PRS-SCZ×childhood trauma on SA in the medial orbitofrontal cortex (β = –0.013, *P*_*uncorrected*_ = .013; Supplementary Table S6): before FDR correction there was a negative association of PRS-SCZ with SA in the medial orbitofrontal cortex in participants exposed to more childhood trauma and a positive association in those who experienced less childhood trauma. More detailed results for the interaction effect are shown in Supplementary Tables S7–S11. Statistics for main effects of the environmental factors are also provided (Supplementary Tables S12–S16).

## Discussion

In this study, we have observed associations between an increased genetic liability for developing schizophrenia and wide-spread cortical differences in the general population, including lower CT in frontal lobe, insula lobe, lateral orbitofrontal cortex, medial orbitofrontal cortex, inferior frontal cortex, and posterior cingulate cortex, as well as reduced SA and CV in the supramarginal cortex and superior temporal cortex. In addition, the associations with reduced SA and CV remained after adjustment for environmental risk factors, indicating that some cortical differences might be driven predominantly by genetic liability for schizophrenia rather than environmental risk factors. Finally, our results suggest that PRS-SCZ and these environmental risk factors might contribute independently to brain abnormalities in schizophrenia.

### PRS-SCZ and Cortical Thickness

Previous studies of the relationship between PRS-SCZ and global CT have been inconsistent, with global cortical thinning^12,30^ or no association^57^ reported. Here, we found no association between PRS-SCZ and global CT, which is unsurprising because schizophrenia-associated genetic variants showed no significant enrichment in mean CT^9^. For regional measures, we replicated significant associations with reduced CT in insula, lateral orbitofrontal cortex, and inferior frontal gyrus, as reported in previous studies in UK Biobank.^5,12,58^ In addition, we observed reduced CT within the frontal lobe, medial orbitofrontal cortex, and posterior cingulate cortex. Reduced CT in these regions has been commonly observed in individuals with schizophrenia^3,17,59–61^ and in individuals at high genetic risk of schizophrenia.^24,59,62^ These regions may also be implicated in the symptoms seen in schizophrenia: the insula is engaged in auditory hallucinations^58^; the orbitofrontal cortex is thought to play a role in negative symptoms^63^; and the frontal lobe has shown associations with cognitive impairment in schizophrenia.^64^

### PRS-SCZ and Surface Area

We found that PRS-SCZ was not associated with global SA. Previous studies in this area have reported inconsistent findings.^12,48,57^ Analyses for regional SA found negative associations within temporal cortices: the supramarginal cortex and superior temporal cortex. This is in line with the significant enrichment of schizophrenia GWAS loci in SA of temporal regions.^9^ Previous studies support structural differences in superior temporal gyrus and inferior frontal cortex as possible biomarkers in schizophrenia.^65,66^ Baseline gray matter reductions in superior temporal and inferior frontal areas are associated with later transition to psychosis.^21^ Additionally, correlations have been observed between superior temporal gyrus atrophy and positive psychotic symptoms, especially auditory verbal hallucinations.^67–69^ Similarly, the involvement of supramarginal and inferior frontal cortex in auditory hallucinations has been extensively documented^70^ and the supramarginal gyrus is associated with delusions of reference and persecutory delusions.^58^ Individual differences in these regions may represent general susceptibility to psychotic symptoms and vulnerability to developing schizophrenia.

### PRS-SCZ and Cortical Volume

Previous findings relating to the relationship between PRS-SCZ and brain volume, have also been inconsistent. Terwisscha van Scheltinga et al.^25^ found PRS-SCZ was significantly associated with smaller total brain volume regardless of disease status, but later studies found different results even within larger samples.^12,26,71,72^ We found no such association. However, we found significant volume reduction in supramarginal cortex and superior temporal cortex. When environmental risk factors were taken into account, an association of PRS-SCZ with lower inferior frontal cortex volume was significant, and associations with both supramarginal cortex and superior temporal cortex were nominally significant. As indicated earlier, this is in line with putative roles for these structures in processing auditory inputs, and their relation to auditory hallucinations in schizophrenia.^66,73,74^

### PRS-SCZ and Subcortical Volumes

Previous studies indicate heterogeneity of findings in subcortical volumes. Although smaller volumes of the pallidum^49^ and thalamus^48^ have been found to be associated with PRS-SCZ, others found no significant association.^5,13,26,57,75^ The largest study to date recently reported associations with smaller hippocampus and larger caudate and putamen volume.^28^ These heterogeneous results may derive from differences in sample characteristics such as age, sample sizes, methods of calculating PRS-SCZ, as well as statistical models and covariates included. Here, we observed no association with the above subcortical volumes and a modest association with the right nucleus accumbens. Smaller nucleus accumbens has been reported in individuals with schizophrenia,^4,5^ as well as in their first-degree relatives.^22,59^ The nucleus accumbens is implicated in numerous neurological and psychiatric disorders, and is a main target of antipsychotic drugs and neurosurgical intervention in schizophrenia.^76,77^ However, it should be noted that the association with the right nucleus accumbens was no longer significant after FDR correction. Further investigation is needed to disentangle the relationship between PRS-SCZ and subcortical volumes.

### Influence of Environmental Risk Factors

When environmental risk factors were included in the model as covariates and participants on antipsychotic medication were excluded, we observed a similar pattern of gray matter reduction; the effects for the supramarginal cortex, superior temporal cortex, and inferior frontal cortex remained significant. Although these environmental risk factors seem to show little impact on brain structure, they are still important considerations. Marsman et al.^78^ examined the relative contributions of genes and environment to mental health and found that familial and environmental factors explained around 17% of the variance, of which around 3% by PRS. Indeed, environmental risk factors, such as cannabis use, childhood trauma, and socioeconomic status, increase the risk for multidimensional symptoms in schizophrenia, including cognitive, affective, negative, and predominantly positive symptoms such as hallucinations and delusions^79–82^ and elevate the risk of developing schizophrenia.^83,84^ Moreover, these environmental risk factors exhibit associations with structural differences in widespread brain regions across frontal, parietal, temporal, and occipital cortex, as well as in subcortical volumes.^46,85–87^ We found evidence of main effects of environment risk factors on certain brain measures, although their associations with brain structures related to PRS-SCZ were weaker compared to those of PRS-SCZ. Future studies on brain abnormalities in schizophrenia should consider the role of environmental risks more closely.

### Correlation Between PRS-SCZ and Environmental Risk Factors

We found that genetic risk for schizophrenia was associated with greater likelihood of using cannabis,^88,89^ experiencing more childhood trauma^90,91^ and higher socioeconomic deprivation, but was not associated with birth weight.^12^ Previous studies on genetic influences on environmental measures demonstrate considerable heritability^92^ and GWASs have found many genetic variants associated with Townsend deprivation index,^93,94^ birth weight^95^, and cannabis use.^96,97^ Other studies also reported genetic correlations between Townsend deprivation index, cannabis use, and schizophrenia.^89,93,96^ As a result, the associations we observed may be due to shared genetic aetiologies.

### Interaction Between PRS-SCZ and Environmental Risk Factors

To our knowledge, only one study so far has reported significant interaction between PRS-SCZ and environmental risk on brain structure: higher PRS-SCZ was associated with lower mean CT in male cannabis users, while there was no significant association in male non-users.^45^ By contrast, studies focused on schizophrenia susceptibility genes especially *COMT* (catechol-O-methyltransferase) and *BDNF* (brain-derived neurotrophic factor) demonstrated interactions with obstetric complications, early life stress, and cannabis abuse,^98,99^ supporting the role of gene–environment interaction. Together with the modest interaction in this study, while preliminary, we recommend that further confirmatory studies in even larger clinical and non-clinical samples should be conducted to improve understanding of gene-environment interactions in schizophrenia.

### Strengths and Limitations

This study has identified associations between PRS-SCZ and multiple brain measures in a large population-based sample. Nevertheless, it should be noted that the variance explained by PRS-SCZ alone is very small (maximum *R*^*2*^ ≤ 0.02%). The use of PRS-SCZ and related biomarkers in clinical practice could be very limited, though it is a promising tool in the translation of genomic discovery to the clinic. In addition, we considered the influence of environmental risk factors for schizophrenia and explored gene-by-environment interactions, and found some weak associations and interactions. Further studies which take these variables into account will need to be undertaken with larger samples. Furthermore, our study was conducted in White British participants aged from 45 to 80, while the peak age for psychosis risk is 15–35.^39^ Including White British participants only meant we were not able to examine the role of some socio-demographic and environmental factors for psychosis, especially those related to ethnicity.^39^ Therefore, we should be very cautious about the generalization of our results to individuals from other backgrounds. Future research extending to diverse populations is warranted to understand the relationship between PRS-SCZ and brain structure. Finally, the environmental factors in this study were limited to childhood trauma, cannabis use, Townsend deprivation index, birth weight, and season of birth. To develop a fuller picture of gene-by-environment interactions, additional studies on other factors such as migration background^39^ are required.

## Conclusion

In summary, this study suggests that gray matter reductions in multiple regions, predominantly in frontal and temporal regions, are neuroanatomical correlates of increased genetic liability for schizophrenia. Further, environmental risk factors such as childhood trauma, cannabis use, Townsend deprivation index, birth weight, and season of birth, appear to contribute very little to these associations, and genetic liability for schizophrenia and these environmental factors might contribute independently to brain abnormalities in schizophrenia. Our findings are meaningful in terms of identifying neuroimaging-based biomarkers for schizophrenia and elucidating the underlying genetic pathophysiology of schizophrenia.

## Supplementary Material

sgab042_suppl_Supplementary_Materials
